# Dissecting the immune evasion and therapeutic resistance mechanisms in EGFR/TP53 co-mutated non-small cell lung cancer: implications for targeted and immunotherapy strategies

**DOI:** 10.3389/fimmu.2025.1652213

**Published:** 2025-08-29

**Authors:** Haiyan Shi, Kun Xu, Xueying Kong, Weining Xie, Yingying Chen, Ding He, Zufu Cheng, Xianshan Huo, Ke Gao, Mingshuang Song, Ning Tian

**Affiliations:** ^1^ Department of Pathology, Guangdong Provincial Hospital of Integrated Traditional Chinese and Western Medicine, Foshan, Guangdong, China; ^2^ Shanghai-MOST Key Laboratory of Health and Disease Genomics, Shanghai Institute for Biomedical and Pharmaceutical Technologies, Shanghai, China; ^3^ Department of Scientific Research, Guangdong Provincial Hospital of Integrated Traditional Chinese and Western Medicine, Foshan, Guangdong, China; ^4^ Shanghai Labway Clinical Laboratory Co., Ltd, Shanghai, China; ^5^ Guangzhou Labway Clinical Laboratory Co., Ltd., Guangzhou, Guangdong, China

**Keywords:** EGFR/TP53 co-mutant NSCLC, single-cell transcriptome analysis, cellular interactions, tumor microenvironment, STAT1/ETS1 axis

## Abstract

**Background:**

Although precision-targeted therapies and tyrosine kinase inhibitors (TKIs) have significantly improved outcomes in non-small-cell lung cancer (NSCLC), patients with EGFR-mutant NSCLC with concurrent TP53 mutations often develop drug resistance and experience poor clinical outcomes. This study aims to investigate the molecular mechanisms underlying this aggressive subtype using single-cell RNA sequencing.

**Methods:**

Formalin-fixed paraffin-embedded (FFPE) tumor samples were obtained from 40 hospitalized NSCLC patients. Somatic mutation profiles were determined using a targeted 23-gene next-generation sequencing (NGS) panel. Four samples harboring concurrent EGFR and TP53 mutations were selected for single-cell transcriptomic profiling using the 10x Genomics platform.

**Results:**

Two dominant malignant epithelial cell populations were identified: C1_EGFR^+^, associated with proliferation and invasion, and C2_STAT1^+^, linked to immunosuppression and drug resistance. These tumor subtypes cooperatively drive CD8^+^ T cell exhaustion through the MDK–(ITGA4+ITGB1), MIF–(CD74+CXCR4), and TGF-β signaling pathways. In addition, antigen-presenting cancer-associated fibroblasts (apCAFs) recruit regulatory T cells via the CCL5–CCR4 axis, collectively establishing an immune-excluded tumor microenvironment. Mechanistically, a STAT1/ETS1-centered transcriptional program regulates the expression of key immunosuppressive (e.g., MDK, MIF, TGFB1) and resistance-associated genes (e.g., ERBB2, JAK2).

**Conclusion:**

These findings reveal a coordinated transcriptional network that promotes immune evasion and therapeutic resistance in EGFR/TP53 co-mutated NSCLC. Targeting the STAT1/ETS1 axis, in combination with EGFR-TKIs or immune checkpoint inhibitors, may provide a novel strategy to overcome resistance and improve patient outcomes. Further validation in larger patient cohorts and functional studies is warranted to confirm these observations and support clinical translation.

## 1 Introduction

Non-small cell lung cancer (NSCLC) is a highly invasive and heterogeneous disease, accounting for approximately 85% of all lung cancers (1, 2). In NSCLC, epidermal growth factor receptor (EGFR) gene mutations are the most common oncogenic driver (3, 4). The use of EGFR tyrosine kinase inhibitors (EGFR-TKIs) has brought significant benefits to the survival of advanced NSCLC patients with EGFR mutations (5). However, clinical observations have found that some patients have poor responses to EGFR-TKIs, suggesting that the biological mechanisms of drug resistance or poor prognosis have not yet been fully revealed (6–8). With the development of next-generation sequencing (NGS), a variety of co-mutations have been found in NSCLC patients with EGFR mutations, among which TP53 mutations are the most common co-mutation type, with an incidence rate as high as 17.3–72.5% (9, 10). Studies have shown that patients with EGFR/TP53 co-mutations have a significantly worse prognosis than patients with EGFR single mutations (11, 12). Therefore, a deep understanding of the remodeling mechanism of cellular composition and molecular characteristics in the EGFR/TP53 co-mutated NSCLC tumor microenvironment (TME) is of great significance for identifying new targets for immunotherapy intervention. TME is composed of immune cells, stromal cells and extracellular matrix (ECM). They interact to provide energy sources for tumors and regulate tumor cell progression through multiple signaling pathways (13). Advances in single-cell RNA sequencing (scRNA-seq) technology have enabled high-resolution characterization of the tumor microenvironment in non-small cell lung cancer (NSCLC) with different mutation types (2). In TME, T cells are an important component of tumor immune microenvironment (TIME) and play a major role in immune surveillance and tumor eradication. According to the different expressions of surface molecules and functional points, T cells can be subdivided into regulatory T cells (Treg), T helper cells (Th), cytotoxic T cells (CTL), etc. During the development of tumors, Treg maintains immune tolerance to self-antigens, allowing tumors to escape and helping to destroy anti-tumor immunity (14, 15). CD8^+^ cytotoxic T play a key role in anti-tumor immunity. However, during chronic infection and tumor progression, continuous antigen stimulation can lead to CD8^+^ cytotoxic T cell exhaustion (TEX) (16–18), resulting in the loss of their effector function and proliferation ability. TEX status has been considered to be one of the important mechanisms of tumor immune escape and immunotherapy resistance (19–22). Therefore, in-depth analysis of the occurrence mechanism of TEX and its dynamic changes in the TME is of great significance for the development of more effective immunotherapy strategies. Cancer-associated fibroblasts (CAFs) are one of the most important stromal cells in TME, playing a multifaceted regulatory role in tumorigenesis, immune escape and treatment resistance (23). In many tumor types, including NSCLC, CAF tumor stemness and chemotherapy resistance are associated with treatment efficiency (24, 25). CAFs can be divided into multiple subtypes based on functional differences, such as myofibroblast-like CAFs (myCAFs) and inflammatory CAFs (iCAFs), which together shape the immunosuppressive microenvironment through ECM remodeling, secretion of growth factors, cytokines and chemokines (26). More and more studies have shown that CAFs can not only indirectly regulate T cell infiltration and function, but may also induce T cells to enter an exhausted state through various mechanisms such as immunosuppressive factors, metabolites or co-stimulatory signals, thereby promoting tumor immune escape (27–29). In addition, there is a class of antigen-presenting CAFs (apCAFs) that can express MHC-II molecules and interact with T cells in an antigen-specific manner (30). However, the origin and role of apCAFs in NSCLC remain unclear.

In this study, we leveraged scRNA-seq to systematically dissect the diversity patterns of the TME in EGFR/TP53 co-mutant NSCLC. We identified distinct tumor cell subpopulations, characterized the immunosuppressive landscape, and uncovered tumor–CAF–T cell interactions that contribute to CD8^+^ T cell exhaustion. Our findings provide mechanistic insights into immune evasion and resistance, and nominate potential targets for precision immunotherapy in this aggressive NSCLC subtype.

## 2 Materials and methods

### 2.1 Ethics and tissue acquisition

A retrospective study was conducted involving 40 Chinese patients with lung cancer who underwent surgical resection at the Guangdong Provincial Hospital of Integrated Traditional Chinese and Western Medicine between January 2024 and July 2024. A total of 40 formalin-fixed, paraffin-embedded (FFPE) tissue specimens were collected for analysis. The study was approved by the Medical Ethics Committee of Guangdong Provincial Hospital of Integrated Traditional Chinese and Western Medicine and conducted in accordance with all applicable ethical guidelines and regulations. Written informed consent was obtained from each patient or their legal guardian prior to any study-related procedures.

### 2.2 DNA extraction from PPFE tissue

Hematoxylin and eosin-stained sections of FFPE tumor biopsies from all samples were reviewed to ensure tumor cell content of >75% when possible, and the tumor area was marked by a pathologist. Genomic DNA was extracted from unstained 10-µm thick FFPE sections using the Kaishuo Biological Technology (Xiamen) Co., Ltd. One-Step Extraction Type FFPE Genomic DNA High-Sensitivity Kit (Cat. No.: RC1102). Extraction was carried out using the Concert 48 Fully Automated Nucleic Acid Extraction System. DNA sample quality was assessed by spectrophotometry using the NanoDrop 2000. During each batch of nucleic acid extraction, one extraction tube without sample was added as a blank control, processed simultaneously with the samples. The concentration range for the blank control should be -5~10 ng/μL. If the blank control concentration abnormally deviates from this range or if amplification is detected in the blank control, the cause of the deviation must be evaluated: If amplification occurs in the blank control, it indicates possible contamination of the blank control tube by sample tubes or inadequate cleaning/disinfection of the workspace during the extraction process. Based on the evaluation results, it is necessary to determine whether a full re-extraction of all samples is required.

### 2.3 Library preparation and sequencing

The target genomic regions were captured using the KM_Solid210–17819 custom probe panel kit from NanoDigmbio (Nanjing) Biotechnology Co., Ltd. This custom panel comprises 17,819 probes, covering approximately 2.13 megabases (Mb) of the human genome. For detailed experimental procedures, refer to the product’s DNA Library Hybridization Capture Operation Guide. Hybridization-based capture library construction was performed according to the QIAseq^®^ FX DNA Library Kit Handbook (Version: January 2020) from QIAGEN. Library sequencing was conducted on the NovaSeq™ 6000 platform. Procedures for library dilution, denaturation, and on-machine sequencing followed the NovaSeq™ 6000 Sequencing System Operation Manual.

### 2.4 Targeted NGS panel-based variant calling pipeline

Raw reads generated from next-generation sequencing underwent quality control using fastp, where low-quality data were filtered out (employing the software’s default parameters) to obtain clean reads. Subsequently, clean reads were mapped to the human reference genome (GRCh37) using the alignment tool BWA to assess the mapping rate. Coverage statistics were calculated using bedtools. Following the generation of duplicate-marked BAM files from the preceding steps, variant calling for SNPs and InDels was performed using GATK, followed by final data quantification. The analysis focused on:Single nucleotide variants (SNVs), Small insertions and deletions (indels), Copy number variations (CNVs) in selected genes, Gene fusions across 23 lung cancer-associated genes (*TP53*, *SLC34A2*, *SDC4*, *ROS1*, *RET*, *RAF1*, *PIK3CA*, *NTRK1*, *MET*, *KRAS*, *KIF5B*, *FGFR3*, *FGFR2*, *FGFR1*, *EZR*, *ERBB2*, *EML4*, *EGFR*, *CD74*, *BRAF*, *ARAF*, *ALK*, *AKT1*).

### 2.5 Tissue dissociation, single-cell transcriptome sequencing

Four NSCLC tumor specimens were obtained from patients who underwent complete surgical dissection. Formalin-fixed paraffin-embedded (FFPE) tissue samples were assessed for RNA quality using the DV200 metric, with only those samples showing a DV200 value greater than 50% considered suitable for further processing. Nuclei were extracted from FFPE tissues following the manufacturer’s protocol (10x Genomics, CG000632). Briefly, 50 µm tissue sections were deparaffinized with xylene and rehydrated through a graded ethanol series. After washing with PBS, the samples were incubated in a dissociation mix (1 mg/ml Liberase in RPMI) at 37°C for 45 minutes with mechanical dissociation. The dissociated samples were then filtered through a 30 µm filter, and the nuclei were resuspended in buffer, stained with AO/PI, and counted using a Countstar analyzer. Nucleus suspensions with a nucleated cell ratio >60%, clumping rate <20%, and a total cell count >40,000 were deemed suitable for library preparation. Library construction was performed using the 10x Genomics Chromium Fixed RNA Profiling protocol (CG000477). Human WTA probes were used for 16-hour hybridization at 42°C. After hybridization and washing, the nuclei were recounted and loaded onto the Chromium X instrument for library construction. The resulting libraries were sequenced on an Illumina NovaSeq platform. The sequencing data (fastq files) were processed by mapping the data to the GRCh38 reference genome using the Cell Ranger toolkit (version 8.0.1).

### 2.6 Single-cell transcriptome data collection and preprocessing

To characterize the compositional and functional status of normal lung tissues and EGFR/TP53 co-mutated NSCLC, we collected single-cell transcriptomic data from public databases for 11 cases of distant normal lung tissues from GSE131907 (31) for inclusion in the analyses. The datasets were all generated using 10x Genomics sequencing technology. For more accurate analysis, we performed quality measurements of the raw gene-cell barcode matrix for each cell based on the following parameters: proportion of mitochondrial genes ≤10%, proportion of hemoglobin genes ≤10%, number of UMIs (nCount_RNA) between 500 and its 95% quartile, and gene count (nFeature_RNA) between 200 and 10,000. After quality control, a total of 61,533 cells were retained, of which 31,672 cells were from normal lung tissues (Normal) and 29,861 cells were from EGFR/TP53 co-mutated NSCLC tissues (Tumor) for further analysis.

### 2.7 Downscaling and clustering analysis

The scRNA-seq data were first normalized by the “NormalizeData” function in the Seurat (version 5.1.0) (32) package, and then scaled using the “ScaleData” function to scale the data. Then, the “FindVariableFeatures” function was used to identify the top 2000 highly variable genes. For dimensionality reduction, we performed principal component analysis (PCA) on the scRNA-seq data using the “RunPCA” function. In order to integrate different datasets and eliminate batch effects, we used the “RunHarmony” function in Harmony (version 1.2.0) (33) for anchor identification and data integration to ensure the consistency of unsupervised cluster analysis in the shared space. The principal components were sorted by the “ElbowPlot” function in the Seurat package, and the top 25 principal components were selected based on the elbow plot. These principal components were then subjected to UMAP/TSNE dimensionality reduction analysis using the “RunUMAP” and “RunTSNE” function to generate a 2D plot for cell visualization. Cell clustering analysis was performed by the “FindClusters” function with a resolution of 0.8. To detect gene expression markers, we also used the “FindAllMarkers” function. Finally, the cell types in the study were annotated using the R package SingleR (version: 2.4.1) (34), the CellMarker (35) dataset, and known cell marker genes.

### 2.8 Identification of malignant cells in EGFR/TP53 co-mutated NSCLC

To distinguish malignant cells within epithelial cells, we assessed somatic large-scale chromosome copy number variation (CNV) scores of individual epithelial cells using the infercnv R software package (version 1.1.1). Raw count matrices, annotation files, and gene/chromosome location files were carefully prepared according to the data prerequisites outlined in the GitHub repository for this project (https://github.com/broadinstitute/inferCNV). During this analysis, normal epithelial cells (“Normal”) were specified as the reference standard for normality, with tumor tissue as the observation group. Using the default parameters (cut.off = 0.1; cluster_by_groups = T), the CNV score was calculated as the cumulative value of CNV regions. Using the copy number score of normal epithelial cells as a reference, cell clusters with CNV, especially those with a significantly higher copy number score than normal cells, were considered malignant.

### 2.9 Cell developmental trajectory analysis

We performed cell developmental trajectory analysis using Monocle2 (version 2.30.1) (36), a widely adopted tool for pseudotemporal inference that assumes high-dimensional gene expression profiles can be projected onto a one-dimensional “pseudotime” axis. Following standard workflows, the data were processed through log-normalization and dimensionality reduction using DDRTree. This enabled the effective visualization of cellular trajectories in a two-dimensional space, revealing a branching structure that reflects potential lineage progression and cell state transitions.

### 2.10 Cellular communication

To infer ligand-receptor interactions between cells, we used the database (CellChatDB) provided by CellChat (version: 2.1.2) (37). The communication probabilities were calculated using the computeCommunProb function, setting type = trimean, trim = 0.1, raw.use = FALSE to use the projection data, thus ensuring the accuracy of the results.

### 2.11 Transcription factor analyses

We used pySCENIC (v0.11.2) (38) to perform transcription factor regulatory network analysis of single-cell transcriptome data to reveal cell type-specific transcriptional regulatory mechanisms. The analysis process consisted of three steps: first, co-expression relationships between transcription factors and their potential target genes were inferred from the normalized expression matrix using the GRNBoost2 algorithm; subsequently, co-expression modules enriched with transcription factor binding sites were screened in combination with the cisTarget motif database to construct high-confidence regulons; finally, the AUCell method was used to calculate regulatory units and activities at the single-cell level.

### 2.12 Statistical analysis and feature-rich analysis

Gene Ontology (GO) annotation and enrichment analyses were performed using clusterProfiler (version: 4.10.1) in R. p.adjust values less than 0.05 were considered significant. All statistical analyses in this study were calculated in R (version: 4.3.3). Data visualization was performed using the communication functions in the R package used for this study or in R using ggplot2 (version: 3.5.1). The flowchart in this article refers to the 10x genomics and bioGDP platforms (39).

## 3 Results

### 3.1 NGS testing and pathological findings in cancer patients

We analyzed NGS panel results from 40 lung cancer patients (26 male, 14 female) over the January to July 2024. Among the 23 lung cancer-associated genes, gene fusions were detected in 2 samples: one with *EML4*-*ALK* fusion and another with *ALK*-unknown gene fusion. Copy number variations (CNVs) were identified in *MET* (1 sample), *PIK3CA* (1 sample), and *EGFR* (4 samples). For SNP and indel mutations: *ERBB2* and *RET* mutations were each detected in only 1 sample; *BRAF*, *MET*, *FGFR2*, and *ROS1* mutations were found in 2 samples each; *PIK3CA* mutations in 4 samples; *KRAS* mutations in 5 samples; *EGFR* mutations in 21 samples; and *TP53* mutations in 20 samples. Notably, 11 samples showed co-occurring *EGFR* and *TP53* mutations. These findings indicate that *EGFR* and *TP53* mutations demonstrate significantly higher prevalence than other genetic alterations in lung cancer tissues. From these, we identified 4 cases with high-quality RNA suitable for single-cell transcriptomic analysis. Detailed mutation profiles, clinical characteristics, and histopathological staining results for these 4 patients are provided in **Appendix Table 1**.

### 3.2 Single-cell transcriptome profiling of EGFR/TP53 co-mutated NSCLC

**Figure 1:**
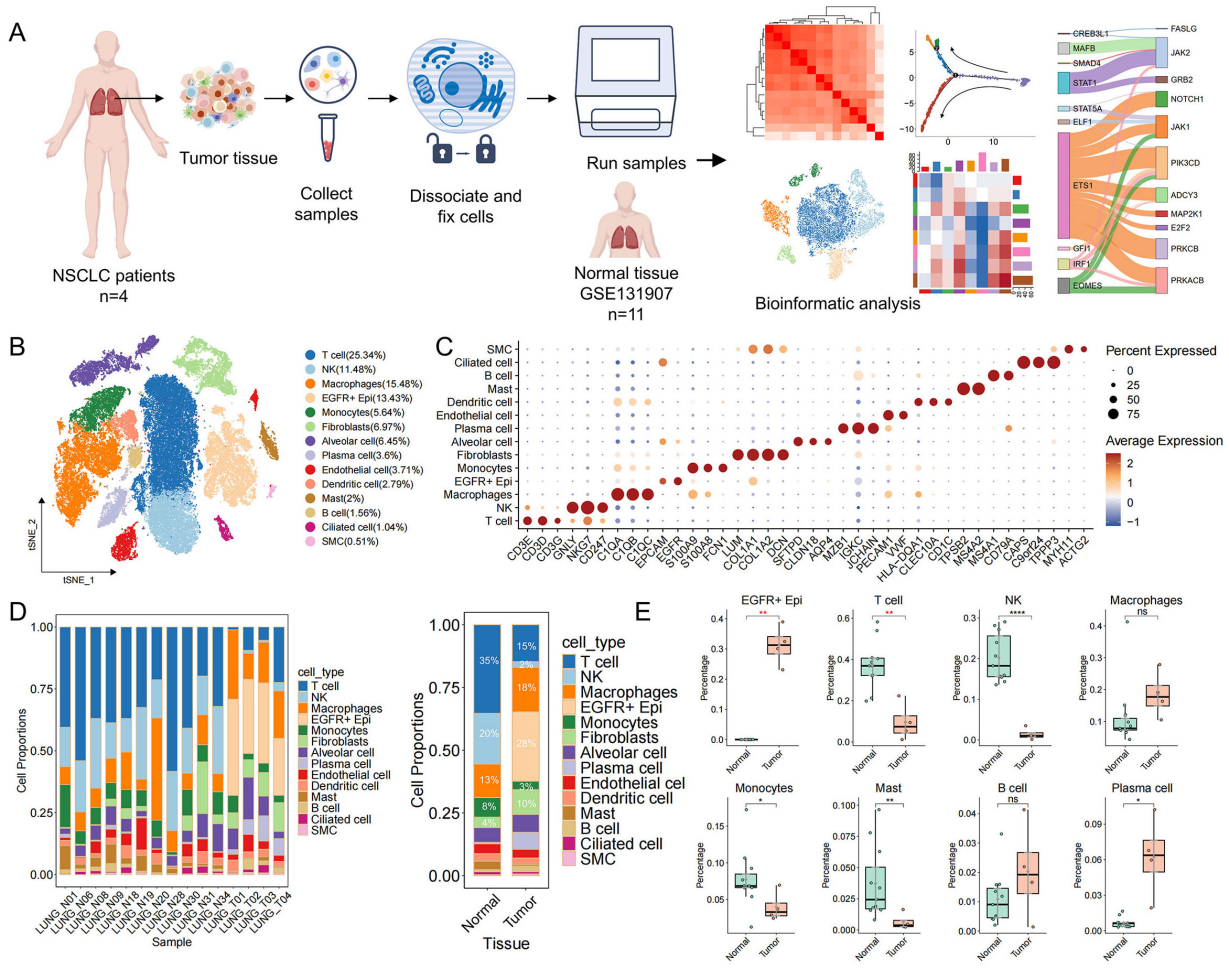
Single-cell transcriptome profile of EGFR/TP53 double-mutated NSCLC. **(A)** Overall framework of the study. **(B)** The t-SNE plots showing different cell type populations and proportions. Each dot denotes one cell; color represents cluster origin. **(C)** Dotplot showing expression markers for 14 cell types. **(D)** Proportional distribution of cells in normal and tumor tissues (left: Sample, right: Tissue). **(E)** Distribution of the proportion of EGFR+ Epi and immune cells in normal and tumor tissues (*p < 0.05, **p < 0.01, ***p < 0.001, ****p < 0.0001).

To delineate the cell type–specific transcriptional landscape of EGFR/TP53 co-mutated non–small cell lung cancer (NSCLC) and its tumor microenvironment (TME), we performed single-cell transcriptome sequencing on tumor specimens from 4 patients harboring concomitant EGFR and TP53 mutations using the 10x Genomics Chromium Fixed RNA Profiling platform. Moreover, to understand tumor-specific changes, we included scRNA-seq data from 11 normal lung tissue samples (**Supplementary Table S1**). After stringent quality control and batch correction, our combined dataset comprised 61,533 cells for downstream analysis (**Figure 1A**). Based on canonical marker genes, cells were categorized into 14 major cell types (**Figures 1B, C**). Within the TME, we identified eight immune subsets—T cells (25.3%), natural killer (NK) cells (11.5%), macrophages (15.5%), monocytes (5.6%), dendritic cells (2.8%), mast cells (2.0%), B cells (1.6%) and plasma cells (3.6%)—three stromal populations—fibroblasts (7.0%), endothelial cells (3.7%) and smooth muscle cells (0.5%)—and three epithelial clusters, including EGFR−positive epithelial cells (EGFR^+^ Epi, 13.4%), alveolar cells (6.5%) and ciliated cells (1.0%). Given that lung adenocarcinoma originates from epithelial cell lineages, we used the R package inferCNV to infer large-scale chromosomal CNVs and used the normal lung epithelial cell spectrum as a reference. We found that tumor-derived EGFR^+^ Epi exhibited pronounced CNV signals across multiple chromosomes (**Supplementary Figures S1A, B**) and were significantly enriched in tumor samples compared to normal controls (**Figures 1D, E**). In contrast, alveolar and ciliated epithelial populations showed minimal CNV alterations and maintained comparable abundances between tumor and normal tissues (**Supplementary Figure S1C**). These findings nominate EGFR^+^ Epi as the predominant malignant compartment in EGFR/TP53 co-mutated NSCLC. Notably, the tumor immune microenvironment exhibited depletion of multiple immune cell populations in tumor samples, suggesting that the expansion of malignant cells may be linked to the reduction of immune cells (**Figures 1D, E**).

### 3.3 Inference of malignant cells in EGFR/TP53 co-mutated NSCLC

**Figure 2:**
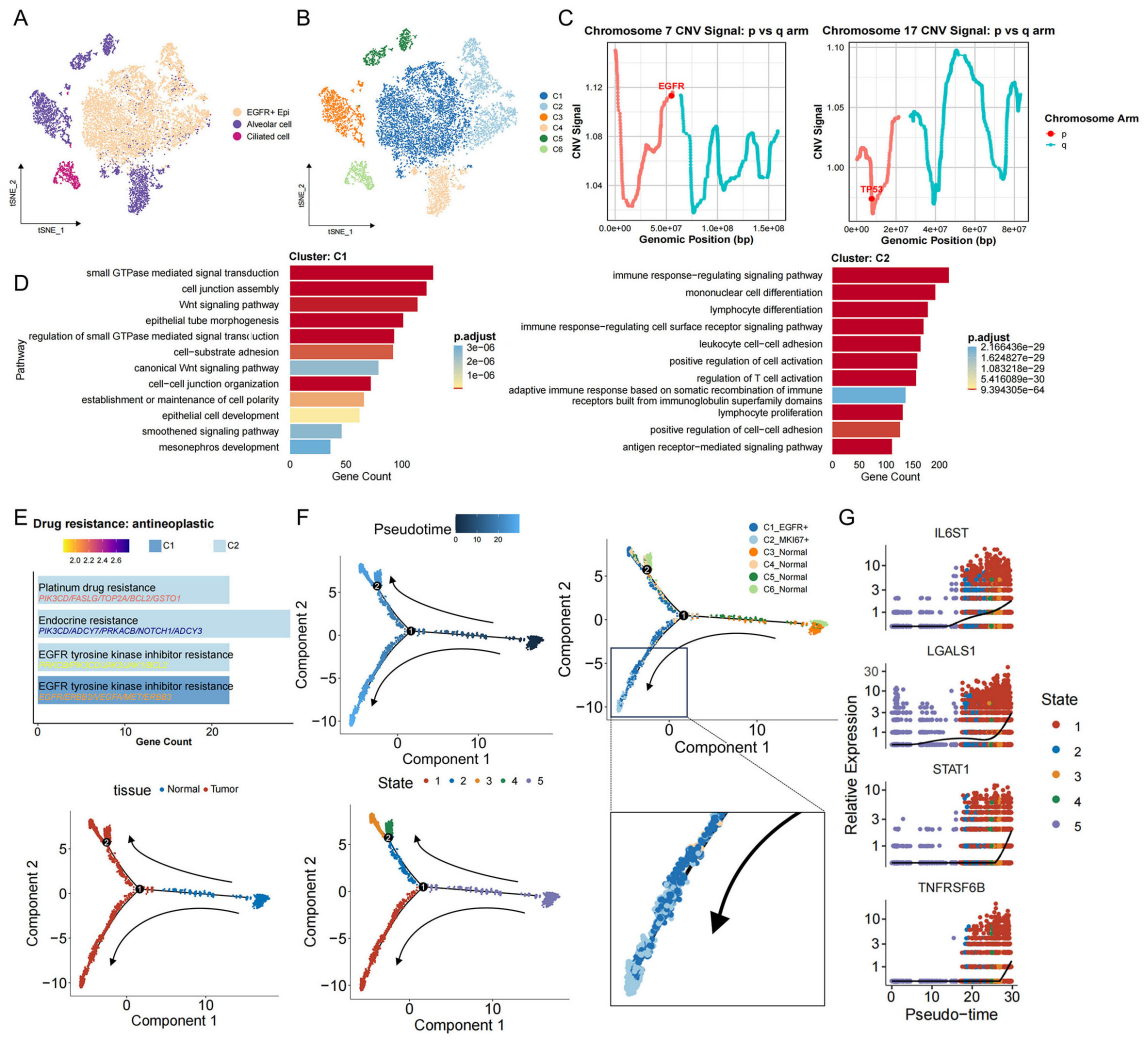
Identification of malignant cells and their developmental trajectories in EGFR/TP53 co-mutated NSCLC. **(A)** The t-SNE plot displays the re-clustering results of the three initially annotated epithelial subgroups. **(B)** The t-SNE plot depicting the re-clustering of newly defined epithelial subgroups. **(C)** Line graph depicting changes in the EGFG gene fragment on chromosome 7 and the TP53 gene fragment on chromosome 17. **(D)** The bar graph illustrates the functional enrichment results for the C1 and C2 groups (p_val_adj < 0.05 & avg_log2FC > 0.25). **(E)** The bar graph presents the enrichment results for drug resistance in the C1 and C2 groups (p_val_adj < 0.05 & avg_log2FC > 0.25). **(F)** The evolutionary trajectory of epithelial cells, including information across different groups (cell type, tissue, and State). Each dot represents a cell and is colored according to its assigned category. Arrows indicate the direction of development. **(G)** Temporal dynamics of immunosuppression-related gene expression. The left end represents the starting point of development, while the right end marks the endpoint.

To elucidate the transcriptional changes and heterogeneity of malignant cells in EGFR/TP53 co-mutated NSCLC, we divided epithelial cells into six subgroups and tumor cell EGFR^+^ Epi into two subgroups (C1 and C2) by unsupervised clustering (**Figures 2A,B**). Both C1 and C2 exhibited marked genomic instability, confirming their identity as dominant tumor compartments (**Supplementary Figures S2A, B**). Focal amplification of EGFR on chromosome 7p was observed in both clusters, accompanied by abnormal expression in the TP53-harboring region on chromosome 17p, supporting the presence of a dual-mutation genotype involving EGFR and TP53 (**Figure 2C**). Notably, large−scale loss of MHC class II loci on chromosome 6 was detected in four samples, indicative of compromised antigen presentation and potential immune−evasion mechanisms (**Supplementary Figures S2C, D**). To characterize tumor cell functional heterogeneity, we compared pathway enrichment between C1 and C2. C1 was selectively enriched for gene sets governing cytoskeletal morphogenesis, cell–cell junction organization, non−canonical Wnt signaling, and small GTPase activity—processes intimately linked to tumor proliferation, invasion, and microenvironmental remodeling (**Figure 2D**). By contrast, C2 showed enrichment for immune−regulatory pathways, lymphocyte proliferation, and cell−adhesion programs, suggesting a role in sculpting an immunosuppressive niche (**Figure 2D**). By further exploring the relationship between tumor cells and treatment resistance. In the ‘Drug Resistance: Anti-tumor Drugs’ module, it was found that C1 were associated with ‘EGFR tyrosine kinase inhibitor resistance’, which aligns with their intrinsic or early resistance to targeted EGFR inhibition. Notably, C2 exhibited a broader spectrum of resistance traits, including ‘endocrine resistance’, ‘platinum drug resistance’, and ‘EGFR tyrosine kinase inhibitor resistance’, underscoring their potential to evade both molecular targeted therapies and cytotoxic treatments (**Figure 2E**). To characterize C1 and C2 cells more clearly, the results of differential gene analysis redefined their names: C1 cells upregulated EGFR (named C1_EGFR^+^), while C2 cells preferentially expressed STAT1 (named C2_STAT1^+^) (**Supplementary Figure S2E**), highlighting their differences in signaling states. Trajectory analysis revealed that C2_STAT1^+^ cells were derived from the C1_EGFR^+^ cell population, marking a transcriptional shift of tumor cells toward a highly immunosuppressive and multidrug-resistant tumor phenotype (**Figure 2F**, **Supplementary Figure S2F**). Along this developmental axis, key immunoregulatory mediators—including IL6ST, LGALS1, STAT1, and TNFRSF6B—were progressively upregulated (**Figure 2G**), implicating C2_STAT1^+^ cells as central drivers of immune evasion, therapeutic resistance, and sustained tumor progression in EGFR/TP53 co-mutated NSCLC.

### 3.4 T cells in the tumor immune microenvironment

**Figure 3:**
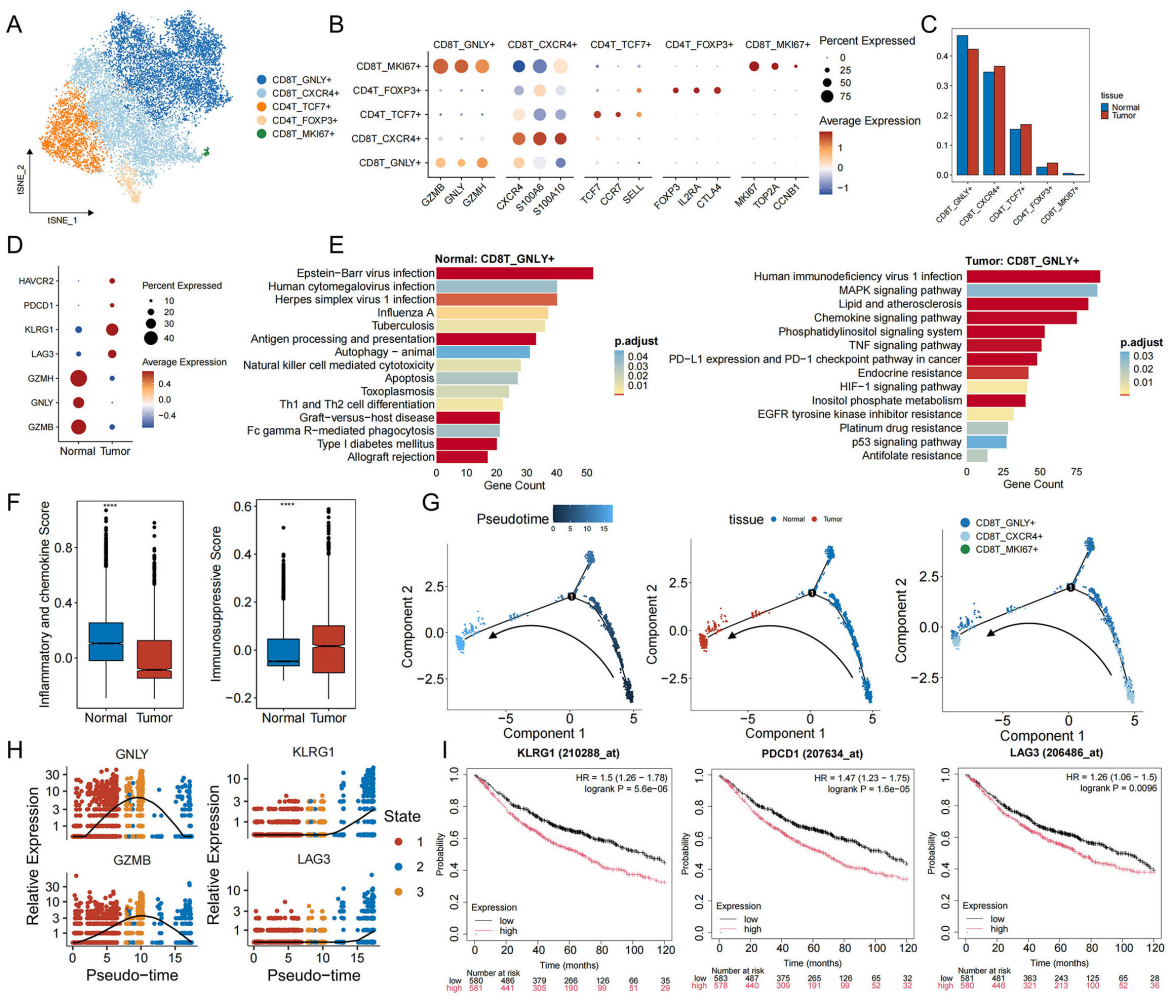
Functional characterization and developmental trajectory of T cells in EGFR/TP53 co-mutant NSCLC. **(A)** TSNE plot of individual T cells. Each dot denotes one cell; color represents cluster origin. **(B)** Dot plots show the expression of selected genes. **(C)** The bar graph illustrates the changes in the proportion of T cell subsets between groups (blue: normal, red: tumor). **(D)** Dot plots displaying the expression of toxicity (GZMB, GNLY, GZMH) and exhaustion markers (LAG3, KLRG1, PDCD1, HAVCR2) in CD8T_GNLY+ cells from normal and tumor tissues. **(E)** The bar graph illustrates the functional enrichment results of CD8T_GNLY+ cells in normal and tumor tissues (p_val_adj < 0.05 and avg_log2FC > 0.25). **(F)** Scoring of pro-inflammatory (IL6, CXCL8, CXCL3, etc.) and anti-inflammatory (IL10, HAVCR2, LGALS9, etc.) markers in CD8T_GNLY+ cells from normal and tumor tissues (****p < 0.0001). **(G)** Developmental trajectory of CD8 T cells, including information on different groups (cell types, tissues). Each dot represents a cell and is colored according to its assigned category. Arrows indicate developmental direction. **(H)** Temporal dynamics of expression of toxic (GNLY, GZMB) and exhaustion genes (KLRG1, LAG3). The left end represents the starting point of development, while the right end marks the endpoint. **(I)** Kaplan-Meier curve showing the survival rate of lung cancer patients with exhaustion genes.

To interrogate the immune landscape accompanying the immunosuppressive tumor cell phenotypes, we next examined the composition and functional states of T cells, which are pivotal for anti−tumor immunity. Notably, the proportion of T cells was markedly reduced in tumor samples relative to adjacent normal tissue (**Figure 1E**). T cells are divided into five different subsets after marker labeling: cytotoxic CD8^+^ T cells (CD8T_GNLY^+^), effector−memory CD8^+^ T cells (CD8T_CXCR4^+^), naive/central CD4^+^ T cells (CD4T_TCF7^+^), regulatory T cells (CD4T_FOXP3^+^), and proliferative CD8^+^ T cells (CD8T_MKI67^+^) (**Figures 3A, B**, **Supplementary Figures S3A, B**). In tumor specimens, the proportion of CD4T_FOXP3^+^ Tregs rose, whereas CD8T_GNLY^+^ cells declined (**Figure 3C**). Functionally, CD8T_GNLY^+^ cells from normal lung tissue exhibited high expression of cytotoxicity markers (GZMB, GNLY, GZMH) and were enriched for antiviral and tumor−lytic pathways. In contrast, CD8T_GNLY^+^ cells from tumor samples upregulated exhaustion markers (LAG3, KLRG1, PDCD1, HAVCR2) and showed enrichment in pathways that shifted from antiviral responses to immunosuppression and signal regulation programs, including PD-L1/PD-1 checkpoints, TNF, MAPK, and HIF-1 signaling pathways (**Figures 3D, E**). Notably, CD8T_GNLY^+^ in tumor samples were also enriched in multiple resistance pathways, including the EGFR tyrosine kinase inhibitor resistance pathway, suggesting that the immunosuppressive microenvironment is promoting or maintaining EGFR-TKI resistance (**Figure 3E**). Immune−function scoring further confirmed this transition: CD8T_GNLY^+^ cells in normal tissue exhibited a high pro−inflammatory and low anti−inflammatory score, whereas those in tumors displayed the inverse pattern (**Figure 3F**) (40, 41), underscoring their acquisition of an inhibited, exhaustion−like state. We then studied the dynamic evolution of CD8^+^ T cells during tumor development. Trajectory analysis results showed (**Figure 3G**) that in the early stages of tumorigenesis, CD8T_GNLY^+^ cells were activated and the expression of GNLY and GZMB increased. However, as the malignancy progressed, these cytotoxic markers gradually weakened, while exhaustion genes (KLRG1, LAG3) steadily increased (**Figure 3H**, **Supplementary Figure S3C**). Clinically, high expression of exhaustion markers correlated with poorer patient prognosis (**Figure 3I**). These data suggest that immunosuppressive signals in the tumor microenvironment drive CD8T_GNLY^+^ cells from a tumor-clearing, proinflammatory state to functional exhaustion, thereby weakening effective antitumor immunity and promoting immune escape and drug resistance.

### 3.5 Cellular communication in the tumor immune microenvironment

**Figure 4:**
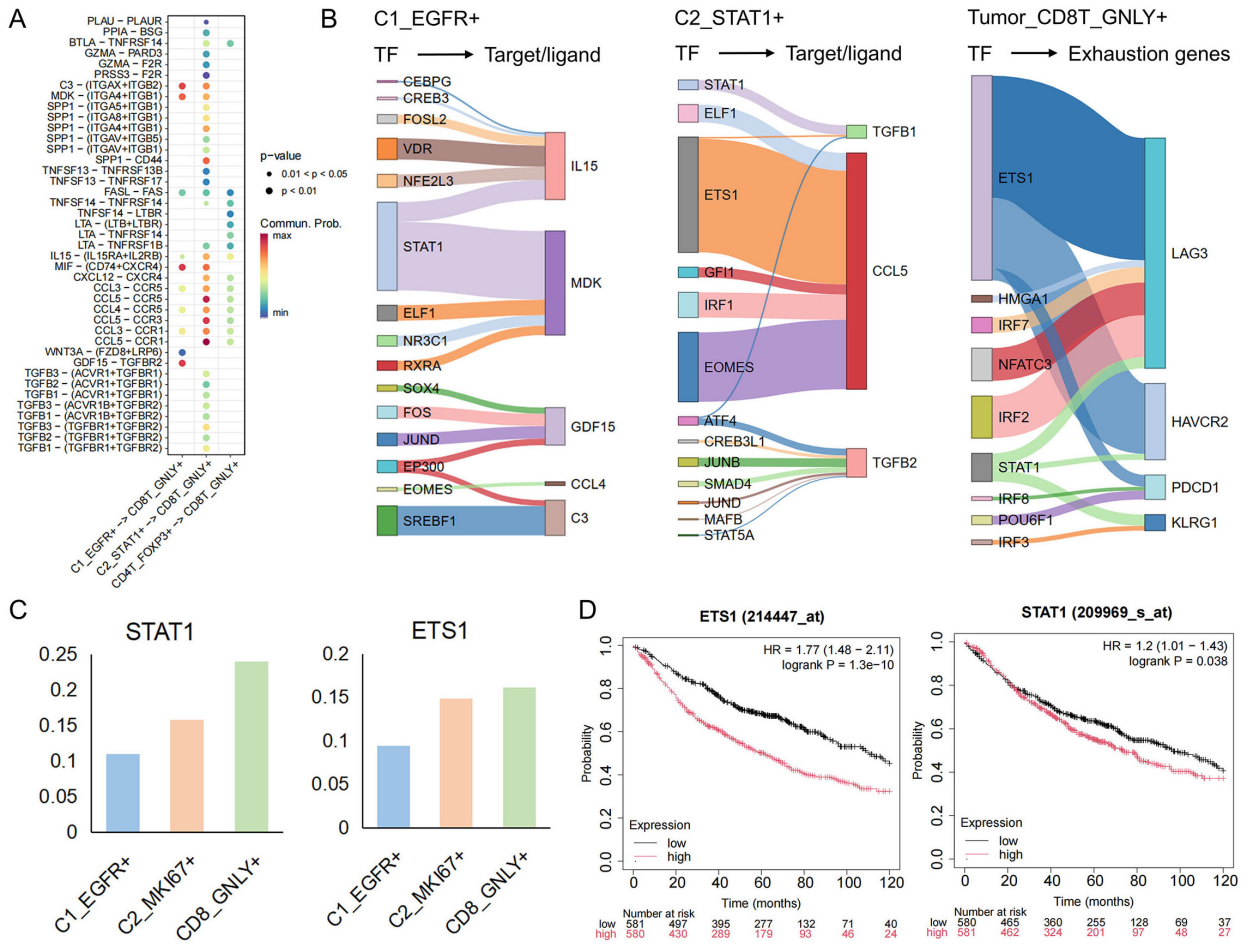
The interaction network between tumor cells and CD8T_GNLY+ T cells. **(A)** Interactions between tumor cells and CD4T_FOXP3+ and CD8T_GNLY+ cells in TME. **(B)** The river plot illustrates the target genes regulated by each cell TF, with the width representing the regulatory weight. The target genes of C1_EGFR+ and C2_STAT1+ are ligands involved in cell communication, while the target genes of CD8T_GNLY+ are associated with exhaustion markers. **(C)** The bar graph shows the expression of transcription factors (STAT1, EST1) in each group. **(D)** Kaplan-Meier curves showing the survival of lung cancer patients with key transcription factors.

Building on our delineation of tumor−cell and T−cell phenotypes, we next sought to unravel the ligand–receptor crosstalk that underlies immune evasion. By integrating single−cell transcriptomes with known ligand–receptor pairs, we constructed an intercellular communication network between malignant subpopulations (C1_EGFR^+^, C2_STAT1^+^) and cytotoxic CD8T_GNLY^+^ cells (**Figure 4A**). This analysis revealed substantially more complex and intimate signaling between C2_STAT1^+^ and CD8T_GNLY^+^ cells than between C1_EGFR^+^ and CD8T_GNLY^+^. Across both tumor clusters, MDK–(ITGA4+ITGB1) and MIF–(CD74+CXCR4) emerged as prominent axes. Midkine (MDK) engages extracellular−matrix receptors to foster an immunosuppressive, pro−angiogenic niche (42), while the MIF–CD74 pathway dampens innate immunity to facilitate tumor progression (43). Notably, C2_STAT1^+^ cells additionally deployed unique chemokine and cytokine signals—CXCL12–CXCR4, CCL5–CCR5/CCR3/CCR1, and TGF−β ligands—that reinforce T−cell exclusion and dysfunction. Tumor−derived CXCL12 and CCL5 are known to recruit suppressive myeloid and regulatory T cells, promote tumor growth, and stimulate neoangiogenesis (44, 45), whereas TGF−β directly attenuates CD8^+^ T cell cytotoxicity and drives exhaustion in chronic inflammatory contexts (46). To identify the upstream drivers of immunomodulatory ligands, we analyzed the activity of key transcription factors. In C1_EGFR^+^ cells, STAT1 mainly regulates the expression of MIF; whereas in C2_STAT1^+^ cells, STAT1 drives the transcription of TGFB1, and ETS1 and EOMES control the expression of CCL5. It was also observed that in CD8T_GNLY^+^ cells, exhaustion-related genes were also co-regulated by ETS1 and STAT1 (**Figure 4B**). Notably, we found that these transcription factors that regulate key ligand signals also regulate pathways associated with drug resistance (**Supplementary Figure S4**). Interestingly, STAT1 and ETS1 activities gradually increased from C1_EGFR^+^ cells to C2_STAT1^+^ cells and further intensified during the subsequent suppression of CD8T_GNLY^+^ cells (**Figure 4C**). Clinically, high expression levels of STAT1 and ETS1 are associated with poor survival outcomes in lung cancer patients (**Figure 4D**). Taken together, these findings suggest the presence of a STAT1 and ETS1-driven transcriptional module in malignant cells that orchestrates a multifaceted immunosuppressive network that not only induces T-cell exhaustion but also enables tumor immune evasion and therapeutic resistance during progression.

### 3.6 Tumor-associated CD74^+^ fibroblasts are associated with the progression of EGFR/TP53 co-mutated NSCLC

**Figure 5:**
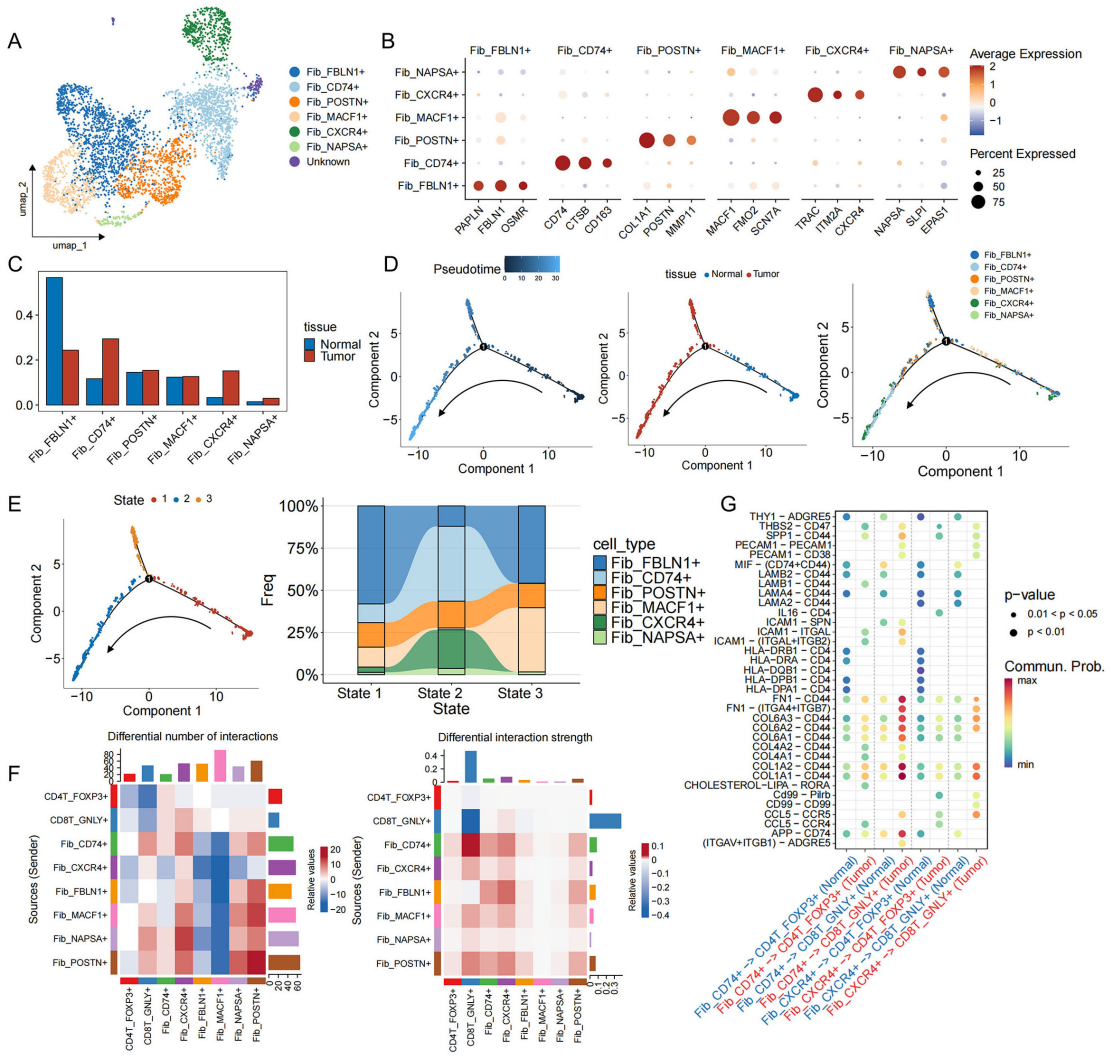
Functional characteristics, developmental trajectories and cell communication of fibroblasts in EGFR/TP53 co-mutant NSCLC. **(A)** UMAP plot of individual fibroblasts. Each dot denotes one cell; color represents cluster origin. **(B)** Dot plots show the expression of selected genes. **(C)** The bar graph illustrates the changes in the proportion of fibroblasts subsets between groups (blue: Normal, red: Tumor). **(D)** Developmental trajectory of fibroblasts, including information on different groups (cell types, tissues). Each dot represents a cell and is colored according to its assigned category. Arrows indicate developmental direction. **(E)** The developmental trajectory of fibroblasts, including three differentiation states. River chart showing the changes in the proportion of fibroblast subpopulations between states. **(F)** Heatmap showing changes in cell communication between fibroblast subsets and CD4T_FOXP3+ and CD8T_GNLY+ cells in tumor samples compared to normal samples. Red indicates a high intensity of interaction between the two cell types in the tumor, while blue indicates a low intensity of interaction. **(G)** Interactions between fibroblast subsets in the TME and CD4T_FOXP3+ and CD8T_GNLY+ cells in normal and tumor samples (blue: Normal, red: Tumor).

CAFs are considered to be key regulators of the malignant environment (47). Six subpopulations of fibroblasts have been identified in EGFR/TP53 co-mutated NSCLC, of which three predominated in tumors: myofibroblastic CAFs (myCAFs: Fib_POSTN^+^), antigen−presenting CAFs (apCAFs: Fib_CD74^+^), and inflammatory CAFs (iCAFs: Fib_CXCR4^+^) (**Figures 5A, B**, **Supplementary Figure S5A**). Relative abundance analysis showed that the proportion of tumor CAFs was increased compared with adjacent normal tissues (**Figure 5C**, **Supplementary Figure S5B**). Time series analysis revealed the state evolution trajectory of the fibroblast population during disease progression (State 1→State 2). As the disease advanced, Fib_FBLN1^+^ fibroblasts—associated with tissue repair and homeostasis gradually transitioned into cancer-associated fibroblasts (CAFs), including Fib_POSTN^+^, Fib_CD74^+^, and Fib_CXCR4^+^ subpopulations (**Figures 5D, E**, **Supplementary Figure S5A**). Comparative ligand-receptor analysis comparing CAF subsets with key T cell populations revealed that Fib_CD74^+^–CD4T_FOXP3^+^/CD8T_GNLY^+^ interactions were significantly enhanced in tumor samples (**Figure 5F**). In normal lung tissue, immune-related axes (MHC-I, CLEC, CypA, LCK) predominate, while in tumors, ECM signals (collagen, FN1, laminin) are significantly upregulated (**Supplementary Figure S5C**). Notably, regarding Fib_CD74^+^–CD4T_FOXP3^+^ MHC-I ligands (HLA-A/B/C) only exist in normal samples, while in the tumor microenvironment, Fib_CD74^+^ promotes the recruitment of CD4T_FOXP3^+^ through the binding of CCL5 to the CCR4 receptor (**Figure 5G**). In addition, two tumor-specific ligand-receptor pairs, SPP1-CD44 and THBS2-CD47, were identified. In other malignancies (**Figure 5G**), SPP1–CD44 signaling exacerbates CD8^+^ T cell exhaustion and fosters resistance to immune−checkpoint blockade (48), while THBS2 promotes epithelial−mesenchymal transition and can induce apoptosis of activated T cells via CD47 (49–53). These data suggest that in EGFR/TP53 co-mutated NSCLC, apCAFs (Fib_CD74^+^) regulate CD8^+^ T cell function by recruiting Tregs, amplifying the immunosuppressive chemokine network, and remodeling the extracellular matrix, thereby cooperating with tumor cells to drive cytotoxic CD8^+^ T cells from an activated state toward exhaustion.

## 4 Discussion

Despite the recent remarkable success of cancer immunotherapy and targeted therapy, it is not effective for everyone, and the mechanism of non-response is not fully understood. Multiple clinical studies have found that TP53 co-mutation is a poor prognostic marker for EGFR-mutated NSCLC and a predictor of poor clinical outcomes for EGFR-TKI treatment (54). In addition, intratumor heterogeneity is an important cause of treatment failure (55). In the past two years, single-cell transcriptomic studies of NSCLC have focused on EGFR or KRAS mutation types (56–58). There is an urgent need to collect single-cell transcriptomic data of NSCLC with TP53/EGFR co-mutation and analyze cell-cell interactions in the tumor microenvironment and their impact on immunotherapy. This study collected samples from NSCLC patients with TP53/EGFR co-mutations and provided a comprehensive landscape of the TME and its adjacent normal tissues at single-cell resolution through scRNA-seq analysis. It was found that tumor cells C2_STAT1^+^ and apCAF (Fib_CD74^+^) would induce the exhaustion of cytotoxic T cells (CD8T_CNLY^+^), leading to enhanced resistance to tumor treatment.

Tumor heterogeneity is a core feature of tumor biology. Its intratumor heterogeneity is manifested by differences in proliferation, invasion, metabolism and immune escape capabilities among cell populations within the same tumor (59, 60) The widespread application of single-cell sequencing has enabled the characterization of tumor heterogeneity at the single-cell level, thereby identifying driver subpopulations, tracking clonal evolution trajectories and revealing interactions between microenvironmental cells (61, 62)​. In this study, based on single-cell CNV inference, we identified C1_EGFR^+^ and C2_STAT1^+^ as the main malignant cell populations in EGFR/TP53 co-mutated NSCLC. Both showed local amplification of EGFR on 7p and abnormalities in the TP53 region on 17p, confirming the existence of dual driver mutation genotypes; and functionally, the C1_EGFR^+^ tumor cells that promote proliferation and invasion dynamically transformed into the C2_STAT1^+^ tumor cells with a highly immunosuppressive and multidrug-resistant phenotype. These findings highlight the key role of intratumor heterogeneity in driving diverse malignant phenotypes, which is one of the main reasons for the failure of targeted therapy and immunotherapy, because certain subpopulations of tumor cells can resist treatment pressure through gene amplification, mutation, or regulation of immune checkpoint molecules (63, 64)​. It suggests that precise intervention targeting the C2_STAT1^+^ subpopulation may break through the dilemma of immune escape and broad-spectrum drug resistance in EGFR/TP53 co-mutated NSCLC.

In the past decade, the study of T cells in the TIME has significantly promoted the development of cancer immunotherapy. Recent single-cell transcriptomic analysis has revealed to some extent the heterogeneity of various types of T cells in tumors and their functional states (65, 66). For example, CCR8 Tregs inhibit the proliferation of CD8 T cells (67, 68). IL-17^+^CD8^+^ T cells, which originate from tissue-resident memory T cells, can differentiate into exhausted T cells and promote tumor progression (69). In this study, we systematically deciphered the heterogeneity and characteristics of T cells and found a substantial transition from toxic T cells to exhausted T cells during tumor progression. It is explained that this transition may be caused by multiple factors. On the one hand, tumor cell C1_EGFR^+^ and C2_STAT1^+^ directly act on CD8T_GNLY^+^ through the MDK–(ITGA4+ITGB1) axis and the MIF–(CD74+CXCR4) axis to promote tumor proliferation. More importantly, C2_STAT1^+^, which has a highly immunosuppressive and multidrug-resistant phenotype, also secretes TGF-β family ligands. TGF-β can directly inhibit the cytotoxic activity of CD8^+^ T cells and drive their exhaustion in the chronic tumor inflammatory environment (46). In addition, TCGA data showed that high expression of MDK, MIF, and TGFBV1 is associated with poor prognosis in patients with non-small cell lung cancer (**Supplementary Figure S4C**). On the other hand, we found that after apCAF (Fib_CD74^+^) lost its MHC II-mediated antigen presentation ability in the tumor microenvironment, it no longer directly activated T cells. Instead, it recruited a large number of CD4T_FOXP3^+^ regulatory T cells by highly expressing CCL5 and binding to the tumor chemokine CCR4 axis, forming a dense immunosuppressive “chemical barrier” (70, 71). Beyond their immunosuppressive function, CD4^+^ T cells may also modulate the activity and recruitment of other immune cells, contributing to the broader immunoregulatory network within the tumor. This apCAF-mediated chemical chemotactic network synergizes with ECM remodeling, not only blocking the physical infiltration of CD8^+^ T cells, but also further strengthening immunosuppression by secreting proinflammatory factors, forming a “multi-layer barrier” to help tumor immune escape and treatment resistance.

Unlike previous studies that focused on the immune checkpoint pathway itself or downstream effects, our study focused on the upstream transcriptional regulation process that drives the generation of key immunosuppressive signals. For the first time, we constructed and systematically characterized a transcriptional regulation module with STAT1 and ETS1 as the core (**Supplementary Figures S4A, B**). This module not only plays a key role in regulating the expression of immunosuppressive-related genes, but also significantly promotes the functional exhaustion of CD8^+^ T cells, tumor immune escape, and the formation of immunotherapy resistance. Although STAT1 has traditionally been considered a classic tumor suppressor, an increasing number of studies have shown that STAT1 has a pro-tumor effect in certain tumor contexts, including colorectal, ovarian, and pancreatic cancers (72–75). We found that the expression level of STAT1 in the treatment response group of patients with NSCLC who received PD-1/PD-L1 immunotherapy was significantly higher than that in the non-response group (p = 0.0448) (**Supplementary Figure S5C**). At present, studies have attempted targeted intervention with STAT1 inhibitors and have shown positive tumor suppressor effects in a variety of solid tumors such as glioblastoma, squamous cell carcinoma, and colorectal cancer (72, 76, 77). ETS1 has been shown to regulate multiple pathways involved in extracellular matrix remodeling, angiogenesis, and immune cell recruitment, thereby promoting tumor invasion and metastasis (78). ETS1 promotes the growth and metastasis of different cancer cell lines. Clinical studies have shown that knockdown of ETS1 inhibits cell transformation and reverses multidrug resistance in breast cancer cells (79). Our analysis found that ETS1 is co-expressed with STAT1 in immunosuppressive tumor subpopulations and promotes the upregulation of immune checkpoint ligands and T cell exhaustion markers.

This suggests that STAT1 is not only a potential prognostic or predictive marker, but also has the potential to become a new target for combined immunotherapy intervention. These findings highlight the STAT1/ETS1 transcriptional module as a critical upstream regulator of the immunosuppressive network in EGFR/TP53 co-mutant NSCLC. These factors may serve not only as biomarkers of treatment resistance but also as potential targets for novel combinatorial immunotherapy strategies.

However, the clinical translation of targeting the STAT1/ETS1 axis still faces significant challenges, including the lack of highly specific small-molecule inhibitors, potential off-target toxicities, and the broad physiological roles of these factors in normal biological processes. Future therapeutic approaches may focus on targeting downstream effectors or tumor-specific regulatory elements to achieve more precise and safer interventions. Additionally, low-dose inhibition of STAT1/ETS1 combined with immune checkpoint blockade could synergistically enhance anti-tumor efficacy while minimizing adverse effects. Moreover, our data indicate that TGF-β signaling and related chemokines, such as CCL5, may serve as more druggable downstream mediators, representing alternative targets for indirect modulation of this immunosuppressive network. We have incorporated these points into the Discussion section to broaden the clinical translational implications of our study.

This study has certain limitations related to sample size and clinical information, including the lack of longitudinal clinical staging data. Nevertheless, we comprehensively evaluated intra-sample and inter-patient heterogeneity using single-cell CNV and transcriptomic analyses, consistently identifying the C2_STAT1^+^ subpopulation across multiple patients, which suggests a level of universality in its underlying mechanisms. Despite these limitations, our findings provide novel insights into the transcriptional regulation of immune evasion in EGFR/TP53 co-mutated NSCLC and highlight the STAT1/ETS1 axis as a promising therapeutic target, laying important groundwork for future mechanistic and translational studies. Moving forward, we aim to expand the sample cohort and utilize immunohistochemistry, flow cytometry, and animal models to further validate and explore the role of this subpopulation across various disease stages and treatment settings. Furthermore, ongoing advancements in spatial transcriptomics and multi-omics technologies will facilitate the integration of spatial data, offering deeper understanding of cell–cell interactions and their dynamic changes over time.

## 5 Conclusion

These findings suggest that the combined effects of C2_STAT1^+^ tumor cells and apCAF (Fib_CD74^+^) cells lead to the exhaustion of CD8 cytotoxic T cells and enhanced drug resistance. We propose that, mechanistically, the key signaling molecules (MDK, MIF, TGF-β, etc.) that cause the failure of CD8 cytotoxic T cells and the genes associated with drug resistance (ERBB2, ERBB3, JAK2, etc.) are regulated by the common transcription factor STAT1/ETS1, forming an upstream transcriptional regulatory network that systematically drives the formation of dual phenotypes of immunosuppression and drug resistance. The network we mapped not only reveals the potential molecular mechanisms of resistance to immunotherapy, but also provides a clear direction for targeted intervention. In the future, combined blockade of the STAT1/ETS1 regulatory axis with immune checkpoint inhibitors, or combined with EGFR-TKI targeted therapy, is expected to break the treatment bottleneck of EGFR/TP53 co-mutated NSCLC and improve patients’ response rate to immunotherapy and overall survival benefits.

## Data Availability

The datasets presented in this study can be found in online repositories. The names of the repository/repositories and accession number(s) can be found in the article/**Supplementary Material**.
