# Supplementary material for: Dissecting the immune evasion and therapeutic resistance mechanisms in EGFR/TP53 co-mutated non-small cell lung cancer: implications for targeted and immunotherapy strategies
Source: Front Immunol. 2025 Aug 29;16:1652213. doi: 10.3389/fimmu.2025.1652213 (PMC12425899; doi:10.3389/fimmu.2025.1652213)
Supplement: Supplementary file 1 [file DataSheet1.docx]

**Supplementary File**


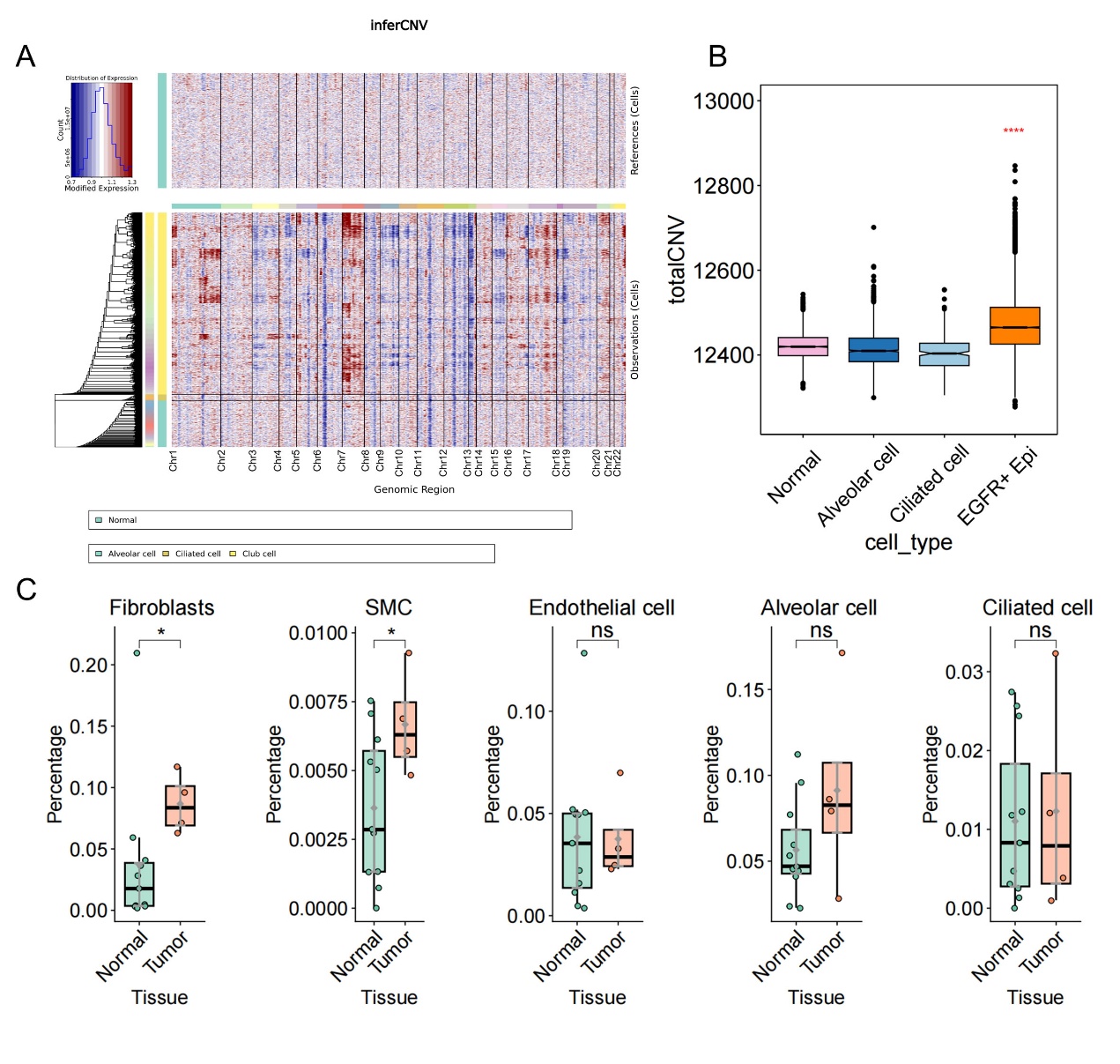


**Fig. S1: Landscape of EGFR/TP53 co-mutant NSCLC tumor cells and their microenvironment.**

(A) The heatmap illustrates the comprehensive CNV profile across epithelial cell clusters. Red and blue shading represent elevated and decreased CNV levels, respectively, compared to normal epithelial cells.

(B) Box plots showing the comparison of CNV scores across different epithelial cell clusters (**** *p* < 0.0001).

(C) The proportional distribution of stromal cells, alveolar cells, and ciliated cells in normal and tumor tissues (**p* < 0.05, ***p* < 0.01, ****p* < 0.001, **** *p* < 0.0001).


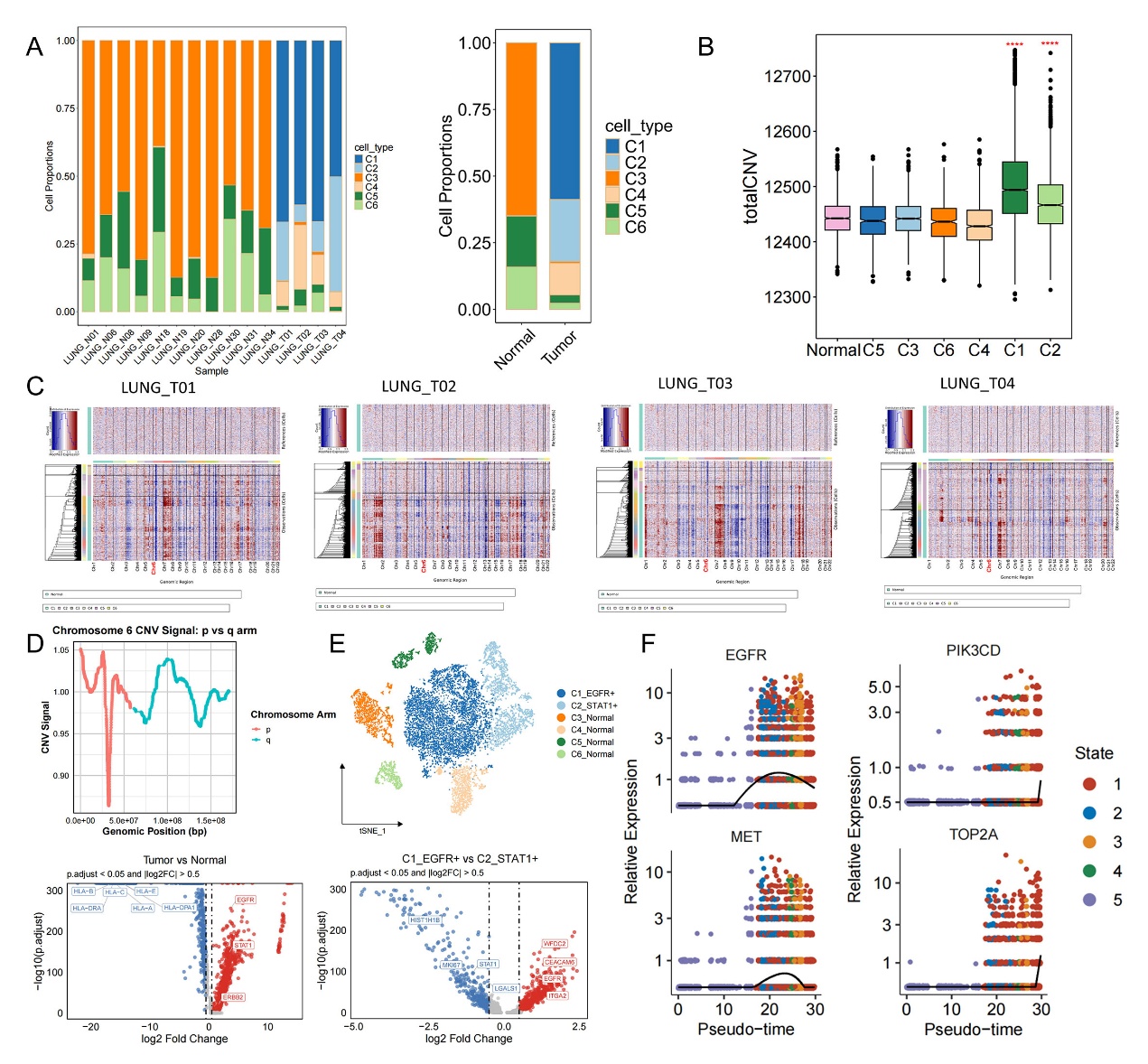


**Fig. S2: Predicting Tumor Cells and Exploring Their Heterogeneity.**

(A) Left: Stacked bar plot showing the distribution of six epithelial cell subclusters (C1–C6) across different samples. Right: Comparison of the overall proportions of the six epithelial cell subclusters between normal and tumor tissues. (B) Box plots comparing total CNV scores among different epithelial cell clusters. "****" indicates *p*-value < 0.0001. (C) Heatmaps displaying CNV profiles of epithelial cells from four tumor samples (LUNG_T01 to LUNG_T04). Red and blue represent elevated and decreased CNV levels, respectively, compared to normal epithelial cells. (D) Top: Line graph showing the CNV signal differences between the p and q arms of chromosome 6. Bottom: Volcano plot showing differential gene expression between tumor and normal epithelial cells (p.adj < 0.05 and |log2FC| > 0.5). (E) Top: t-SNE plot illustrating the clustering of epithelial cells, colored by identified subclusters (C1_EGFR+, C2_STAT1+, and different normal cell groups). Bottom: Volcano plot showing differential gene expression between C1_EGFR+ and C2_STAT1+ clusters (p.adj < 0.05 and |log2FC| > 0.5). (F) Scatter plots showing the relative expression dynamics of EGFR, PIK3CD, MET, and TOP2A genes along pseudotime trajectories in epithelial cells. Different colors represent distinct pseudotime states.


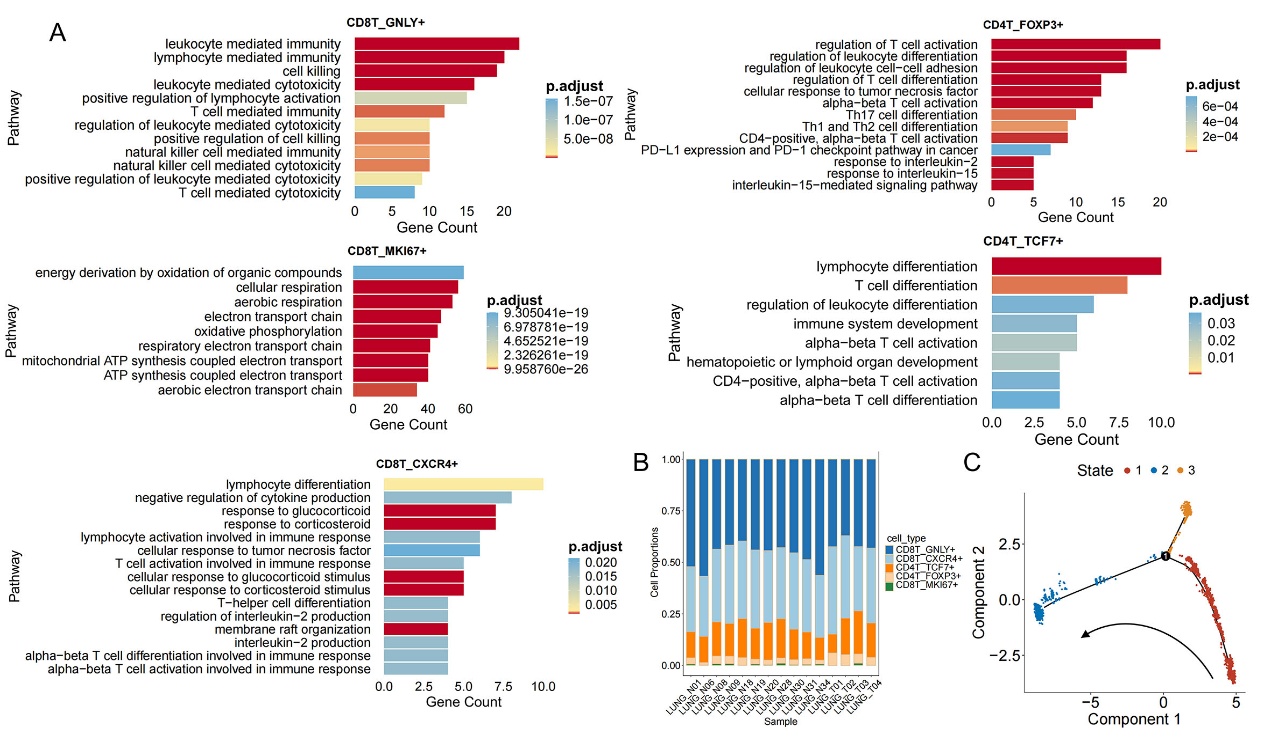
**Fig. S3: Functional analysis of T cell subsets.**

(A) Bar plots illustrating functional enrichment of T cell subsets. Each bar graph represents the enriched biological functions (e.g., CD8T_GNLY+, CD4T_FOXP3+, CD8T_MKI67+) associated with a specific T cell subset. Enrichment analysis was based on genes with adjusted p-values < 0.05 and log2 fold change > 0.25. (B) Stacked bar plot showing the distribution of five T cell subclusters across different samples. (C) The plot depicts the dynamic progression of CD8⁺ T cells across pseudotime. Each dot represents an individual cell, colored by its assigned developmental state. Arrows indicate the inferred direction of cellular differentiation.


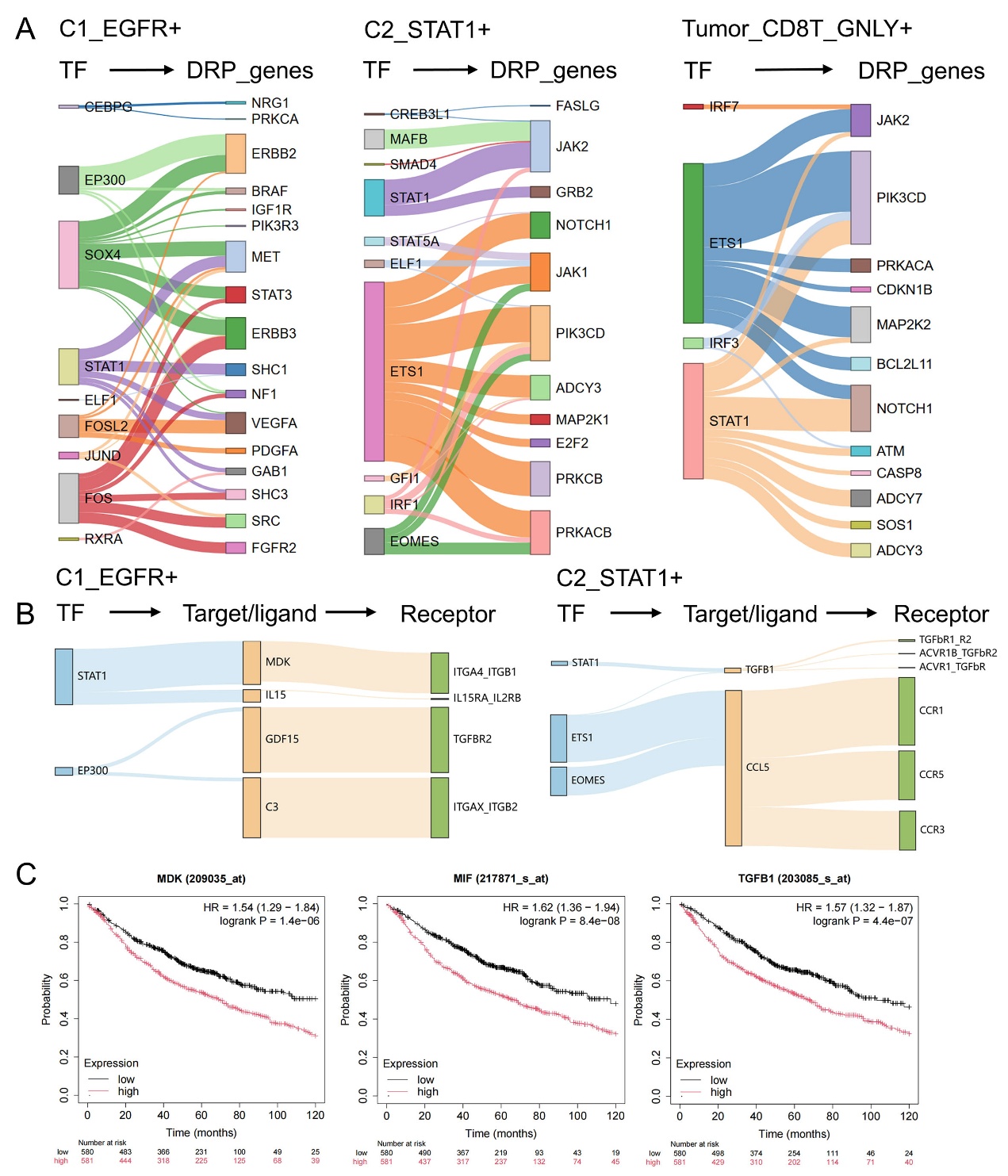
**Fig. S4: Transcription Factor Regulatory Modules of Genes**

(A) Transcription Factor Regulatory Modules of Genes Enriched in the Drug Resistance Pathway (DRP) in C1_EGFR+, C2_STAT1+, and CD8T_GNLY+ T Cells within Tumors. (B) STAT1/ETS1 transcription factor regulatory module. (C) Survival analysis of related ligands.


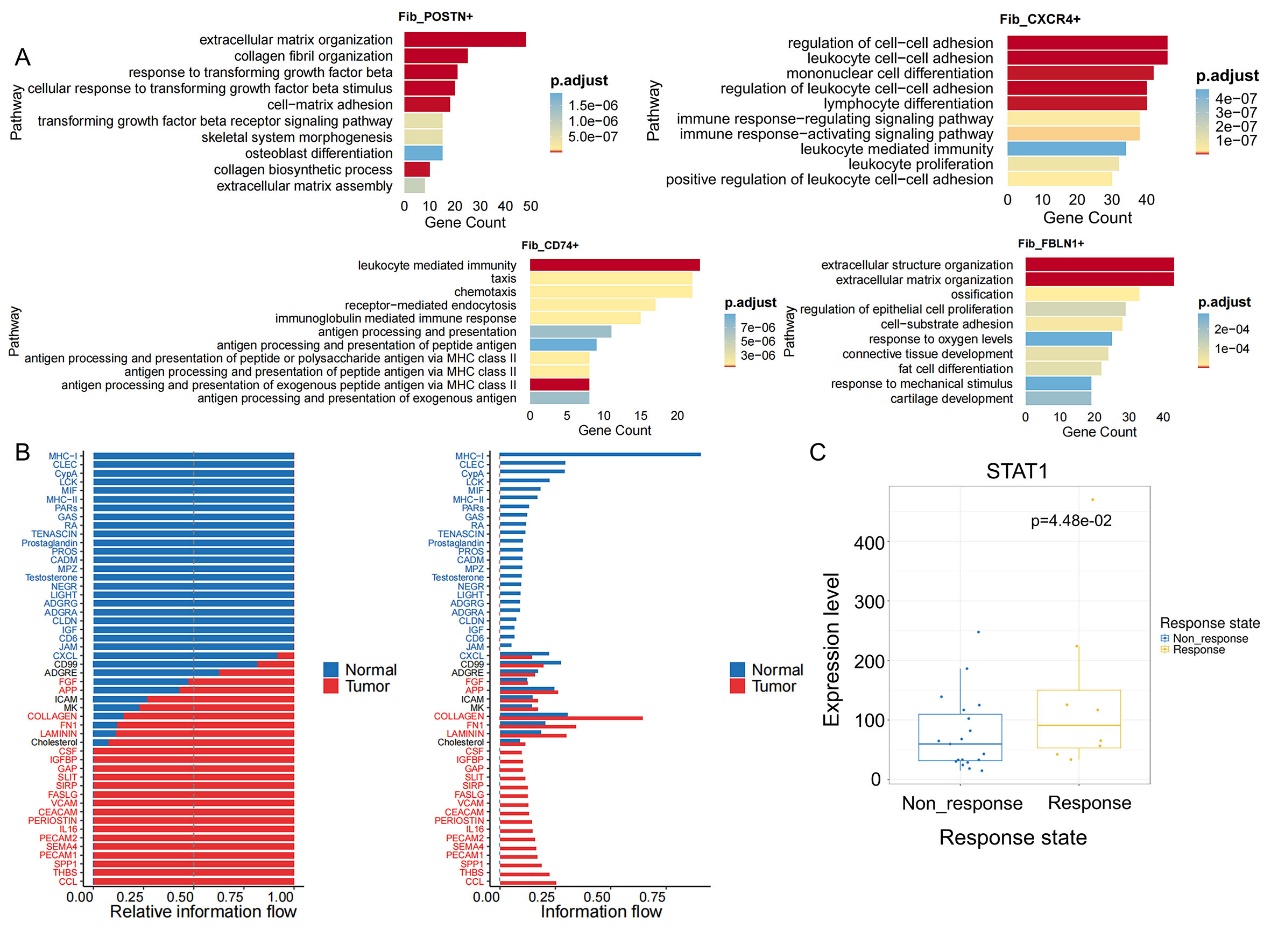


**Fig. S5: Functional analysis of Fibroblasts subsets.**

(A) Bar graph showing functional enrichment of fibroblast subsets. Each bar represents an enriched biological function associated with a specific CAF (e.g., Fib_FBLN1+, Fib_CXCR4+, Fib_CD74+). Enrichment analysis was based on genes with adjusted p-value < 0.05 and log2 fold change > 0.25. (B) Stacked bar plot showing the distribution of six fibroblast subclusters across different samples. (C) Interaction Signaling Pathways Between Fibroblast Subsets and CD8T_GNLY+ and CD4T_FOXP3+ T Cells in Normal and Tumor Tissues. The blue pathways represent more active signaling pathways in Normal, and the red pathways represent more active signaling pathways in Tumor.
